# Estimation of equilibrium times and maximum capacity of adsorption of heavy metals by *E. crassipes* (review)

**DOI:** 10.1007/s10661-019-8032-9

**Published:** 2020-01-25

**Authors:** Uriel Fernando Carreño Sayago, Yineth Pineros Castro, Laura Rosa Conde Rivera, Alexander Garcia Mariaca

**Affiliations:** 1 Fundacion Universitaria Los Libertadores, Cra. 16 #63a-68, Bogotá, Cundinamarca Colombia; 2 Jorge Tadeo Lozano University, Cra. 4 #22-61, Bogotá, Cundinamarca Colombia

**Keywords:** Cellulose, Heavy metal, Isotherm, Biofilter

## Abstract

Cellulose emerges as an alternative for the treatment of water contaminated with heavy metals due to its abundant biomass and its proven potential in the adsorption of pollutants. The aquatic plant *Eichhornia crassipes* is an option as raw material in the contribution of cellulose due to its enormous presence in contaminated wetlands, rivers, and lakes. The efficiency in the removal of heavy metals is due to the cation exchange between the hydroxyl groups and carboxyl groups present in the biomass of *E. crassipes* with heavy metals. Through different chemical and physical transformations of the biomass of *E. crassipes*The objective of this review article is to provide a discussion on the different mechanisms of adsorption of the biomass of *E. crassipes* to retain heavy metals and dyes. In addition to estimating equilibrium, times through kinetic models of adsorption and maximum capacities of this biomass through equilibrium models with isotherms, in order to design one biofilter for treatment systems on a larger scale represented the effluents of a real industry.

## Introduction

The contamination of rivers, lakes, and wetlands among others is increasingly evident; the lack of corporate responsibility and the lack of alternatives for water treatment is one of the main problems that leads to the serious affectation of these water resources with different pollutants like heavy metals.

At present, different technologies are available for the removal of heavy metals using treatment systems such as advanced chemical oxidation, membrane separation, photocatalysis, oxidation, and adsorption (Crini 2005), but these treatment systems are usually very costly. Among all the available methods for the treatment of industrial effluents, adsorption remains the most widely used methods in the treatment of wastewater due to a low capital cost; in addition, it can eliminate most types of contaminants and facilitate regeneration (Hokkanen et al. 2013; Putro et al. 2017a, b).

The process of adsorption of heavy metals with cellulose is interesting due to its abundant availability and its great potential in removing heavy metals present in water, demonstrating in experimental processes in the treatment of water (Chitpong et al. 2017; Putro et al. 2017a, b; Lu et al. 2017; Pillai et al. 2013; Saman et al. 2017). The presence in the cellulose of hydroxyl (OH), amino (NH_2_), and carboxyl (C=O) groups facilitate the adsorption of heavy metals through cation exchange (Lin et al. 2018; Anirudhan et al. 2016).

Nowadays, almost all adsorbents are developed for the removal of heavy metal ions and based on the interaction with the functional groups that are present in the adsorbents (Hokkanen et al. 2014; Yu et al. 2013; Ruan et al. 2016).

Different types of biomass have the ability to adsorb heavy metals, for example, chitosan (Zhou et al. 2014) coconut residues (Pillai et al. 2013), banana (Saman et al. 2017), bacterial cellulose (Baldikova et al. 2017; Jin et al. 2017), plant cellulose from camphor leaf (*Cinnamomum camphora*) (Wang et al. 2018), and cellulose from *Eichhornia crassipes* (Gupta and Balomajumder 2015; Hadad et al. 2011; Swain et al. 2014; Borker et al. 2013).

The biomass of *E. crassipes* with a large amount of hemicellulose (30%) and cellulose (35%) in it is vegetal structure is an alternative for the treatment of waters contaminated with heavy metals, and it is very abundant in contaminated wetlands and lagoons. (Abdel-Fattah and Abdel-Naby 2012; Ganguly et al. 2012; Bronzato 2016; Rahman et al. 2016; Park et al. 2017).

The aquatic plant, *E. crassipes*, grows in wetlands and is usually contaminated with organic matter, which tends to its rapid expansion in the surface where a layer is created that does not allow oxygen access to the interior of the water; therefore, they cease to exist microorganisms vital to the ecosystem. (Martínez et al. 2013; Saraswat and Rai 2010; Feng et al. 2017; Liu et al. 2018; Mohammed et al. 2018; Adornado et al. 2017; Rani et al. 2017; Mahunon et al. 2018).

In experimental processes at laboratory scale, the dry and crushed biomass of *E. crassipes* has been contacted with water contaminated with different heavy metals, with removals that do not exceed 75%. (Lin et al. 2009; Atehortua and Gartner 2003; Sarkar et al. 2017; Ibrahim et al. 2012; Yi et al. 2016).

One way to optimize the process of removing heavy metals is through the chemical or physical transformation of the biomass of *E. crassipes*. Various modifications, such as processes, modification of cellulose with carbon disulfide (xanthogenate), and the impregnation of iron (Fe) (III), have been used to improve the sorption capacity of the dead biomass of *E. crassipes* (Ri et al. 2017; Nagarajan et al. 2017; Anirudhan et al. 2015). To evaluate the removals and the adsorption capacities, the isotherm models have been used, finding through this the maximum adsorption capacity of the different biomass transformed from *E. crassipes*, specifically the Langmuir isotherm. (Ammar et al. 2014,2014).

In addition, adjustments to second-order kinetic adsorption models have been reported in different experiments, finding with this model the saturation equilibrium time of the different biomasses and concluding that the chemisorption is the form by which there was the exchange of the metals heavy in biomass transformed from *E. crassipes.* (Mohanty et al. 2006; Chisutia and Mmari 2014; Liu et al. 2018; Vijetha et al. 2014; Gogoi et al. 2017; Yi et al. 2016; Lin et al. 2012).

The objective of this review article is to provide a discussion on the different mechanisms of adsorption of the biomass of *E. crassipes* to retain heavy metals and dyes. In addition to this objective is to estimate the equilibrium times through kinetic models of adsorption and maximum capacities of this biomass through equilibrium models with isotherms, in order to design biofilter systems on a larger scale that represented the effluents of a real industry.

## Characteristics of *E. crassipes* as adsorbent

The macrophyte *E. crassipes*, also known as “water hyacinth”, is a floating vascular plant (macrophyte) of fresh water native to South America (Brazil and equatorial region). Its stems and leaves are shape of by air-filled sacs allowing it to permanently suspend on the surface of the water; it presents both sexual and asexual reproduction and prevails mainly in tropical and sub-tropical water (Chuang et al. 2012; Wang et al. 2015a, b).

*E. crassipes* is considered as an invasive species, due to its adaptability to a wide type of ecosystems, which considerably affects the natural balance of aquatic systems (lagoons, lakes, wetlands, among others) and feeds mainly on the concentration of nutrients of effluents from agro-industry, deforestation, or insufficient water treatment. *E. crassipes* has significant amounts of cellulose and hemicellulose in its plant morphology, which is because it has been used in many phytoremediation and bioenergy projects (Kumar et al. 2017; Mishima et al. 2008; Magdum et al. 2012; Baldeón et al. 2017; Wu et al. 2015).

Among its main attributes is its very low content of lignin present in its structure, in contrast to the high content of cellulose, per unit volume of dry matter, making it easily degradable. Table 1 shows different characterizations of the *E. crassipes* for phytoremediation purposes or other treatments.

**Table 1:** Composition of the biomass of *E. crassipes*

Lignin (%)	Cellulose (%)	Hemicellulose (%)	Others* (%)	Reference
1.1	17.3	24.7		Chuang et al. (2012)
4.1	19.7	27.1		Mishima et al. (2008)
3.5	18.2	48.7	13.3	Magdum et al. (2012)
1.1	17.3	24.7		Lay et al. (2013)
11	31	27	10	Tan et al. (2008)
11	27	27	10	Zhou et al. (2009)
12	36	42		Balasubramaniana et al. (2012)

*Ashes

**Fig. 1:**
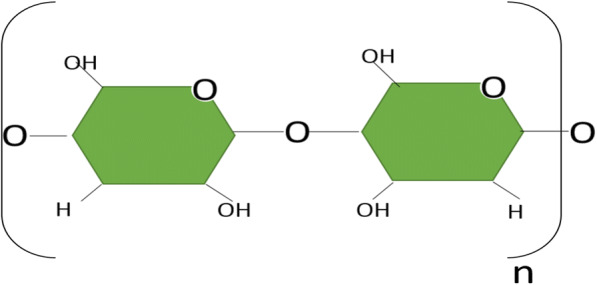
Vegetable cellulose from *E. crassipes* modification (Pillai et al. 2013)

The dry and ground biomass of the plant *E. crassipes* are being used for the creation of biological filters and to treat waters contaminated with heavy metals. Atehortua and Gartner (2003) dried and ground the roots of *E. crassipes*, placing them in contact with a solution of zinc (II), chromium (VI), and cadmium (II), with initial concentrations of 100 mg/L of each pollutant, removing less than about 70% of each heavy metal. Figure 1 shows the molecular structure of *E. crassipes* cellulose in which the functional groups (OH) involved in heavy metal removal processes are highlighted, since they have a negative charge, which favors the adsorption of these contaminants (Tan et al. 2008). Initial concentrations of 100 mg/L of each pollutant remove less than about 70% of each heavy metal. Figure 1 shows the molecular structure of *E. crassipes* cellulose in which the functional groups (OH) involved in heavy metal removal processes are highlighted, because they have a negative charge, which favors the adsorption of these contaminants.

Ibrahim et al. (2012) characterized physicochemically the cellulose structure of the plant *E. crassipes* and characterized the mathematical models of adsorption of this biomass to remove cadmium (cd), conducting a batch experiment, removing a total of 75%, with an initial concentration of 100 mg/L. A cation exchange is reported between the hydrogen bonds of the functional groups of the plant with the cadmium, favoring the process of removal. The mechanism of adsorption of uranium by dried and crushed biomass of *E. crassipes* has also been reported. The results showed that the adsorption of uranium (VI) was highly dependent on pH, reaching the best values ​​at pH 5.5. The adsorption of U (VI) progressed rapidly with an equilibrium time of 30 min and adjusted to a second order kinetics (Yi et al. 2016).

## Modification of cellulose and mechanisms of adsorption of the *E. crassipes* biomass

With the aim of improving the adsorption of heavy metals, chemical or physical modifications of the cellulose of *E. crassipes* can be made. The properties of this biomass, such as its hydrophilic or hydrophobic character, elasticity, water absorbance, capacity of adsorption or ion exchange, resistance to microbiological attack, and thermal resistance, are generally modified by physical or chemical treatments (Anirudhan and Shainy 2015; Hokkanen et al. 2015; Zhang et al. 2016).

Generally, heavy metals are cations (possess a positive charge) because they have lost electrons, represented with an oxidation state (+) (Kobielska et al. 2018), for example, magnesium (II), aluminum (III), lead (II), zinc (II), copper (II), mercury (II), cobalt (II), arsenic (III) and (V), chromium (III), and (VI) among others (Nguyen et al. 2018), which means that these heavy metals need electrons to complete their last level of energy. These electrons are what are putting into play during the chemisorption process (Tortora et al. 2018).

## Modification of cellulose with carbon disulfide (xanthogenate)

**Fig. 2:**
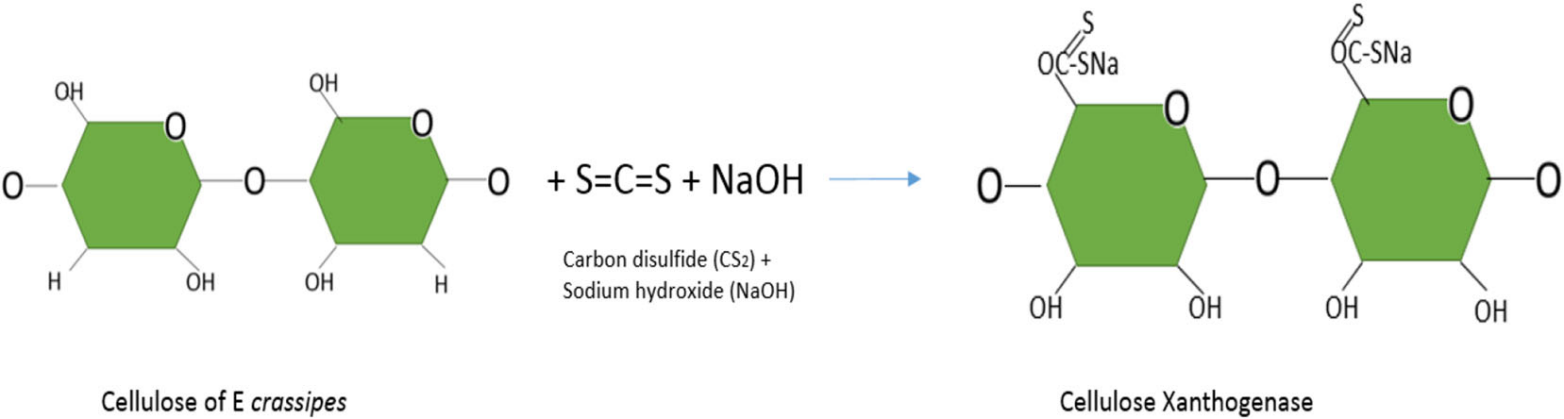
Representation of xanthogenase cellulose production, based on Zhou et al. (2011)

The cellulose xanthogenate is one of the cellulose transformations of *E. crassipes* to improve the adsorption performance of heavy metals; this compound is produced from dry and ground biomass, mixed with sodium hydroxide (NaOH) to eliminate the lignin, creating alkaline biomass; then carbon disulfide (CS_2_) is added (Tan et al. 2008; Den et al. 2012; Zhou et al. 2011). Figure 2 shows the union of sulfur disulfide (CS_2_) in the bond of plant cellulose, along with sodium (Na) responsible for the exchange with metal ions; it can be seen that for each glucose, it adheres the disulfide and sodium.

**Fig. 3:**
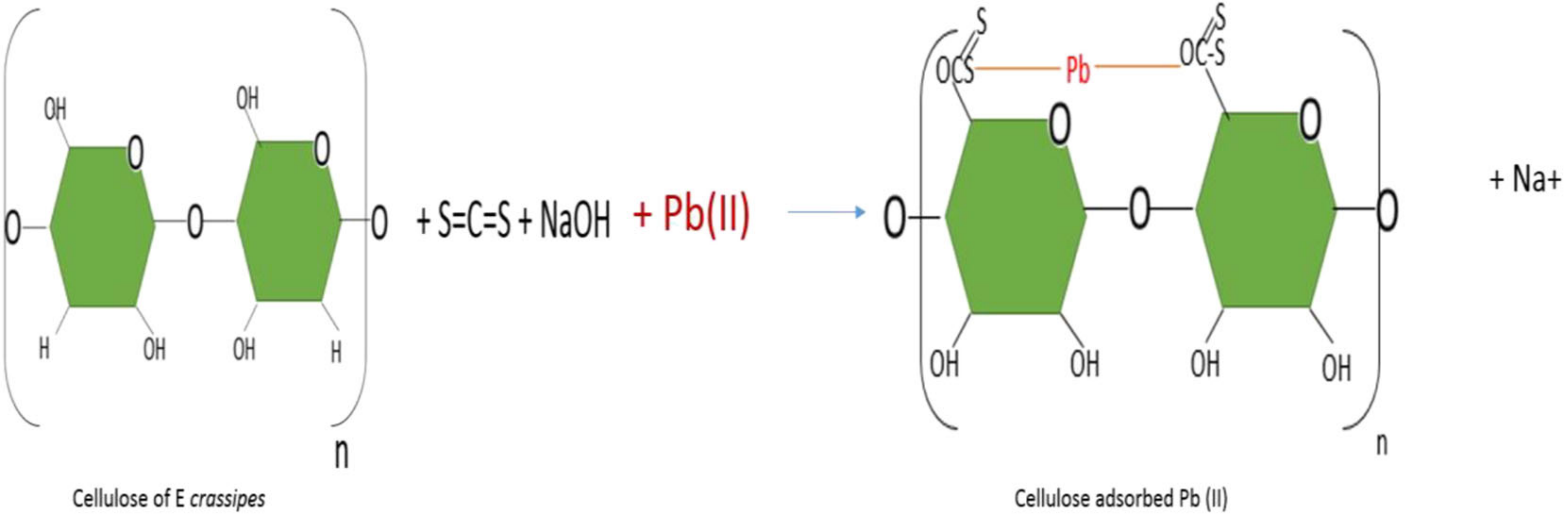
Adsorption of lead by cellulose xanthogenase, based on Wang et al. (2018)

One of the examples is the use for the removal of lead (II), with cellulose xanthogenase reported by Wang et al. (2018), represented in Fig. 3. The lead remains adhered to cellulose xanthogenase in a cation exchange chemisorption process.

The pH has a significant effect on the adsorption capacity of cellulose xanthogenase. Ranges of pH 2.0–7.0 were studied. When the pH is in the range of 2.0–5.0, there is a competitive state with the H+ to form a chelate complex with the cellulose xanthogenase groups and among the heavy metals. This is because the percentage of protonation of the groups of cellulose xanthogenase can increase and the number of active sites for the absorption of Pb (II) decreases with decreasing pH (Jin et al. 2017; Zhou et al. 2011; Pillai et al. 2013; Wang et al. 2018).

As the pH increases, the cell surface change of xanthogenase becomes more negative and the adsorption capacity towards metal ions increases substantially, due to the electrostatic attractions between the opposite charges ions. However, when the pH reaches eight, the large amount of OH anions can lead to the metal oxide formation. Under this condition, the mechanism of adsorption of heavy metals at a high pH would be complicated and it would be difficult to distinguish between adsorption and precipitation of metal ions; therefore, the optimum pH for the adsorption of metal ions is between five and eight (Huang et al. 2018; Jin et al. 2017; Stoica-Guzun et al. 2016).

## Modifications of cellulose with iron

**Fig. 4:**
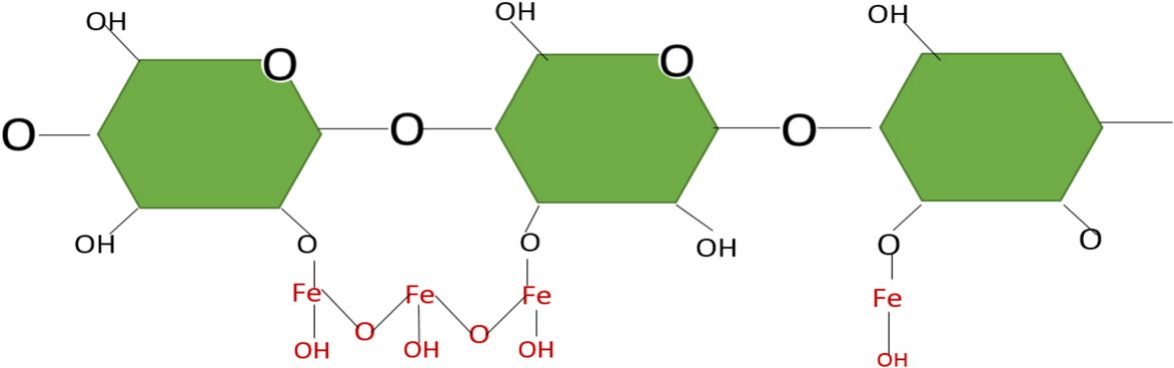
Cellulose reaction with iron chloride based on Lin et al. (2016)

The impregnation of iron (Fe) (III) through iron chloride to the surface of *E. crassipes* was used for the adsorption of different heavy metals and dyes. The (Fe) (III) reacts with hydratable hydroxyl cellulose, forming iron hydroxides (FeOOH); these are responsible for the cation exchange with heavy metals; the metal ions enter the interior of *E. crassipes* with FeOOH, exchanging with protons of hydroxyl groups. The ionic interaction was mainly responsible for the adsorption of As (III), As (VI), and Cr (IV) (Wei et al. 2017; Lin et al. 2018). Figure 4 shows the reaction of cellulose with iron chloride (FeCl), forming iron hydroxides (FeOH).

**Fig. 5:**
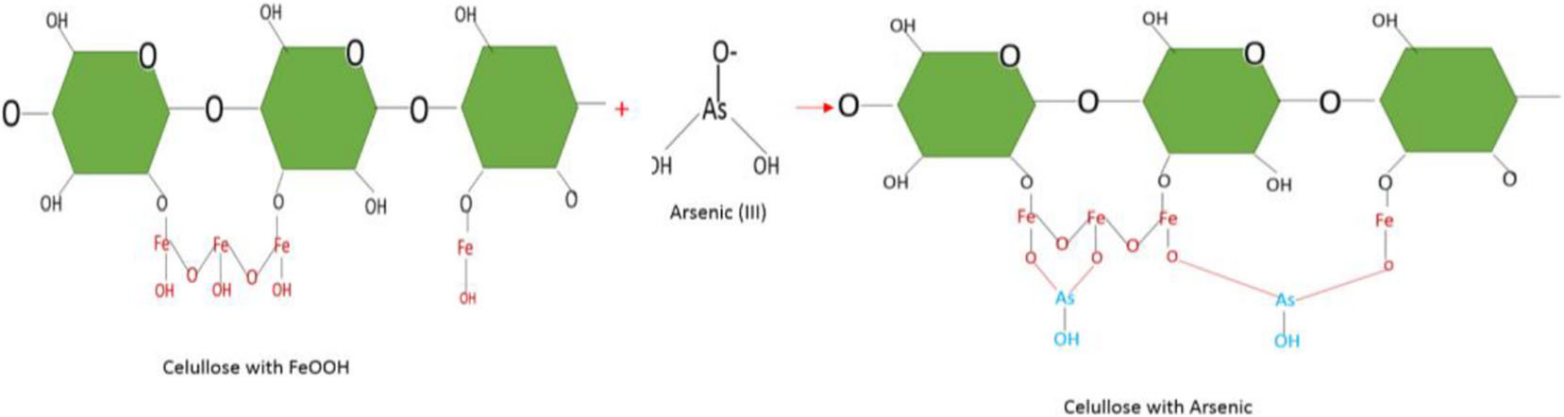
Cellulose reactions with Fe-adsorbing arsenic (III) based on Lin et al. (2016)

**Fig. 6:**
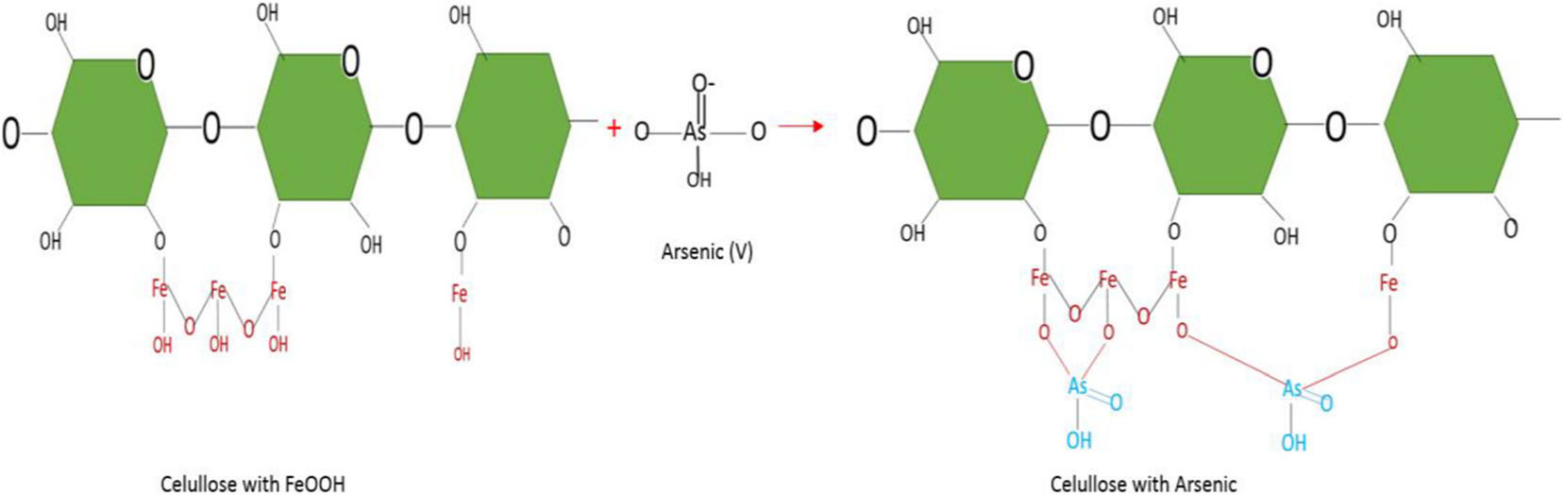
Cellulose reactions with Fe-adsorbing arsenic (V) based on Lin et al. (2016)

The elimination of metal ions species by Fe was explained by their adsorption on the surface of the corrosion product of the original Fe particles. Therefore, when contacted with a solution, Fe (0) gradually oxidizes plant cellulose allowing the ions of different heavy metals to form internal sphere complexes with oxidized sites (Hokkanen et al. 2015). In Figs. 5 and 6, the adsorption of Arsenic (III) and (V) can be seen.

Through FTIR spectrophotometry, it was shown that hydroxyl groups are the main ones involved in the Arsenic bond, forming a coordination complex between cellulose and arsenic with the participation of the functional group (OH) (Chen et al. 2012).

**Fig. 7:**
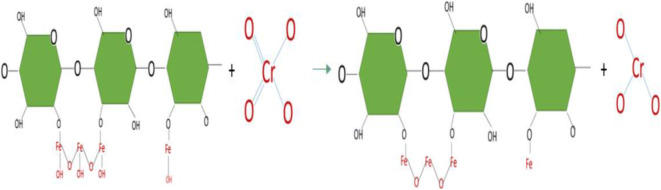
Reaction of reduction of Cr (VI) to Cr (III) of cellulose modified with iron based on Park et al. (2006)

The adsorption on the cell surface of chromium ions is a product by the physicochemical interaction between the metal and the functional groups present on the surface of the cell of the microorganism (Dhankhar and Hooda 2011). The Cr (VI) in aqueous solution was removed by dead biomass from two mechanisms. The first mechanism consists of direct reduction, in which the Cr (VI) was reduced to Cr (III) in the aqueous phase by contact with the electron donor groups present in the biomass such as the (OH) and (NH_2_) groups. In Fig. 7, the reduction of Cr (VI) to Cr (III) by cellulose modified with iron can be seen.

**Fig. 8:**
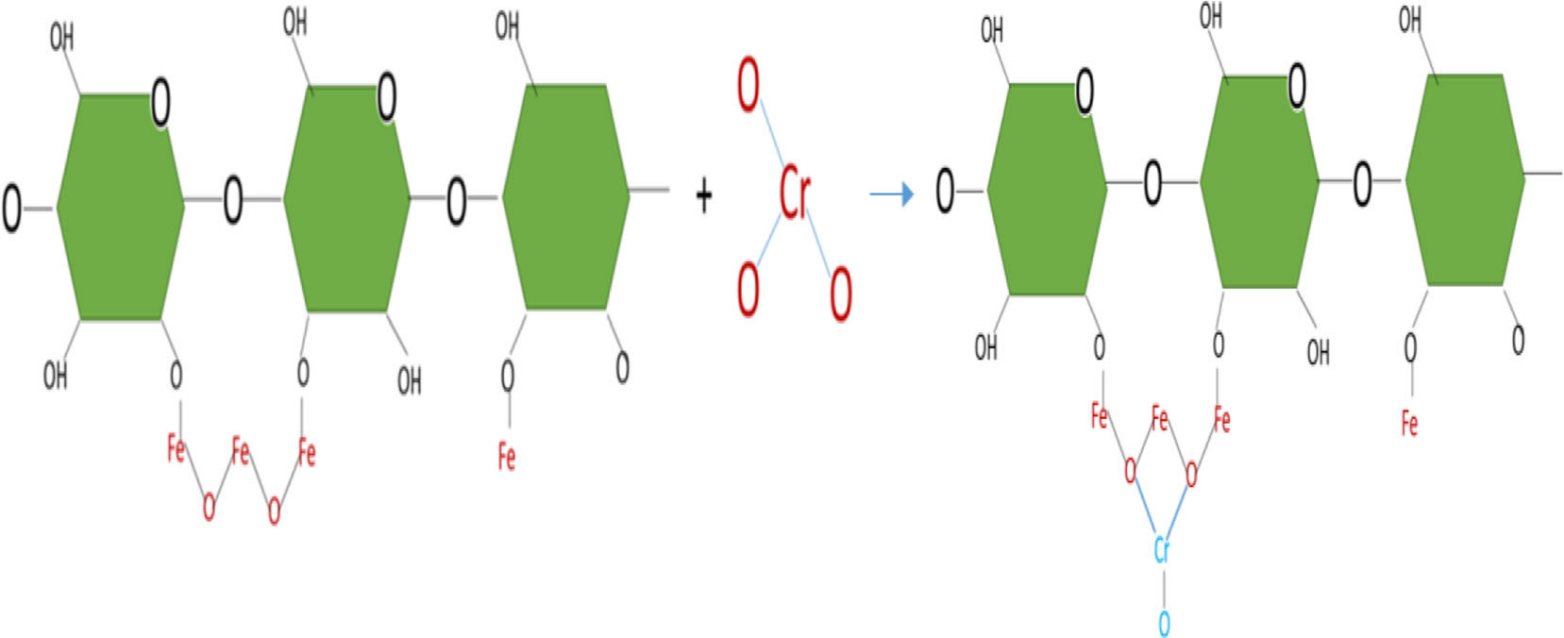
Reaction of chromium (III) adsorption by iron-modified cellulose based on Park et al. (2006)

The second mechanism is the indirect reduction, in which three stages are identified: (1) the binding of the anionic species of Cr (VI), with the functional groups present on the surface of the biomass positively charged; (2) the reduction from Cr (VI) to Cr (III) by the adjacent electron donor groups, and finally, (3) the release of Cr (III) ions in the aqueous phase due to the repulsion between the positively charged groups and the ions Cr (III), or by the complexation of Cr (III) with adjacent groups capable of binding Cr. In conclusion, the mechanism of removal of Cr (VI) by biomaterials is a combined mechanism between adsorption and reduction (Park et al. 2005; Park et al. 2006). In Fig. 8, the process of adsorption of Cr (III) by iron-modified cellulose can be seen.

The effect of pH on the cellulose impregnated with iron in the adsorption of heavy metals is a material of many researches to achieve the optimum level of adsorption, due to the exposure to water at different pH. The surface of the iron oxide develops charges on the surface and, by adsorbing the metal ions, completes the coordination layers with the OH groups, which bind or release H+. Under neutral and acidic conditions (less than 8), the OH2+ and OH forms on the surface are dominant and responsible for the selective binding of the molecular and ionic forms of the metal ion species (Hokkanen et al. 2015; Liu et al. 2018).

The effects of pH on the adsorption of Cu (II) and Pb (II) on modified bacterial cellulose were evident. When the pH is low, large numbers of hydrogen ions compete with Cu (II) and Pb (II) for the adsorption site of −NH_2_. With the increase in pH, the amino groups are gradually de-activated and their chelation capacity with Cu (II) and Pb (II) is improving, which causes the increase of the adsorption capacity (Niu et al. 2017; Huang et al. 2018). In addition, the abundant hydroxyl in bacterial cellulose was ionized under high pH conditions and can show a strong electrostatic attraction with metal ions in the solution (Jin et al. 2017).

Ammar et al. (2014) developed a composite with biomass of *E. crassipes* and chitosan, creating microspheres with sodium tripolyphosphate solution (TPP). *E. crassipes* cellulose fibers are embedded in the chitosan matrix. In the experimental treatment, these areas removed about 95% lead (II) with initial concentrations of 100 mg/L. In the gelled cellulose compound of *E. crassipes* and chitosan, the lead was adhered through a cation exchange of chemisorption. The ionic interaction was mainly responsible for the adsorption of Pb (II) in the spheres.

Another composite using cellulose from *E. crassipes*, chitosan and titanium oxide (TiO), has been investigated, experienced removals above 90% Congo red as a function of pH. There is an electrostatic interaction between the OH-negatively charged hydroxyl groups of cellulose fibers of *E. crassipes* together with the NH (III) anion of chitosan; the TiO2 binds to this compound and forms a chelating network; this new compound increases the cation exchange capacity (El-Zawahry et al. 2016).

With *E. crassipes* and polyvinyl alcohol, hydrogels have been created, becoming a more versatile adsorption material in wastewater treatments for the elimination of dyes and heavy metals due to the unusual chemical molecular structure, because it has a chelating group of amide (-CONH_2_) which is an active site where metals are housed (Rahchamani et al. 2011; Zhou et al. 2014). These compounds have a high porosity (99.56%) and low density (0.0065 g/cm) (Yin et al. 2017). The polyvinyl alcohol (PVA) helps in the removal of heavy metals, since this polymer has too many electrons, potentially activating the exchange with heavy metals, due to its positive charge of these pollutants (Ajitha et al. 2017).

## Models of adsorption isotherms

Adsorption models have been studied, and different mathematical models have been created by isotherms. An adsorption isotherm is a curve that describes the phenomenon that regulates the retention of a substance from aqueous media to a solid phase at variable temperature and pH (Zhou et al. 2009; Ariff et al. 2017).

The Langmuir isothermal model assumes that adsorption occurs at specific homogenous sites in the biomass of *E. crassipes* and was successfully used in many monolayer adsorption processes (Yap et al. 2017: Sastry and Rao 2017; Djehaf et al. 2017). The non-linear and linearized forms of the Langmuir isotherm equation is as follows:

1 $$ \mathrm{qe}=\frac{\mathrm{QoBCe}}{1+\mathrm{BCe}\ } $$

qe: the capacity in the adsorption equilibrium in a given time (mg of metal/g of *E. crassipes*)
Ce: concentrations of heavy metal in equilibrium (mg/L)
Qo: maximum capacity (mg/g of biomass).
B: Langmuir constant isotherm parameter related to affinity of binding sites

2 $$ \frac{1}{\kern0.5em \mathrm{qe}}=\frac{1}{\mathrm{Qo}} + \left(\frac{1}{\mathrm{Qo}\mathrm{B}}\right)\frac{1}{\mathrm{Ce}} $$

Rl is also known as the separation factor, given by:

3 $$ \mathrm{Rl}=\frac{1}{1+\mathrm{BCe}\ }. $$

The Langmuir model represents the experimental data of heavy metal adsorption on *E. crassipes* better than the other adsorption models. (Yi et al. 2016; Lin et al. 2012; El-Zawahry et al. 2016; Li et al. 2013; Lin et al. 2018; Ammar et al. 2014; Zhang et al. 2015; Sumanjit et al. 2015; Feng et al. 2017; Gogoi et al. 2017). These evidences indicate that the adsorption of heavy metals on the adsorbents of *E. crassipes* are monolayer adsorption. The modification of *E. crassipes* gave significant effects on the adsorption performance of heavy metals, because more functional groups of modifying agents was bound on the surface of *E. crassipes*, and the surface became more homogeneous. Table 2 shows a summary of each adsorption experiment that was adjust to the Langmuir isotherms, along with the parameter count, the *R*^2^, and the capacities of each biomass to retain heavy metals.

**Table 2:** Langmuir isotherms adjusted in heavy metal treatment with *E. crassipes*

Author	Contaminate	Transformations of the biomass	Isotherm of adsorptions of Langmuir			
			b	qm(mg/g)	*R*^2^	pH
Yi et al. (2016)	Uranio	No	0.28	142	0.99	6–7
Lin et al. (2012)	Arsenic As (V)	No	0.1	2.5	0.9987	8
	Arsenic As (III)	No	0.1	1.1	0.9987	8
Li et al. (2013)	Copper (2)	No	0.2	32	0.998	5
	Chrome (3)	No	0.3	33	0.998	2
Liu et al. (2018)	sulfocloropiridazina (SCP)	No	2.4	0.163	0.998	
Lin et al. (2018)	Arsenic As (V)	FeOOH	0.3	9.61	0.9987	8
	Arsenic As (III)	FeOOH	0.3	5.5	0.9987	8
Ammar et al. (2014)	Lead (2)	Esferas gelificadas con quitosano	0.9	312.5	0.989	6
Zhang et al. (2015)	Chrome	Biochar	0.137	64	0.96	3
Feng et al. (2017)	Cadmium	Biochar	0.7	70.3	0.94	5
El-Zawahryaty et al. (2016)			0.28	27.6	0.983	5
Tan et al. (2008)	Copper	Celulosa xantogenato	0.33	262	0.97	5
Zhou et al. (2011)	Copper	Celulosa xantogenato	0.44	302	0.88	4
Deng et al. (2012)	Lead	Celulosa xantogenato	0.55	252	0.98	8
Chen et al. (2012)	Cadmium(II)	Quitosano, *crassipes* y TiO_2_	0.44	256	0.98	7

The Langmuir model shows (mg) the maximum amount of heavy metal retained on 1 g of biomass *E. crassipes* raw or transformed, obtaining the maximum capacity (qm) in (mg/g) as a source of future designs of waste treatment systems sewage water.

The microspheres, containing *E. crassipes*, chitosan, and sodium tripolyphosphate (TPP), were analyzed for the adsorption of lead (II); this new adsorbent resulted in a maximum loading capacity of 312.5 mg of Pb (II), where they adsorbed a gram of gelled spheres of *E. crassipes* and chitosan. The adsorption data followed the Langmuir model. The cellulose from *E. crassipes* exhibited an adsorption capacity of only 12 mg/g of Pb (II) (Ammar et al. 2014).

The maximum adsorption capacity of arsenic (As) (III) and (V) by *E. crassipes* with FeOOH was determined through the Langmuir model, resulting in 5.5 and 9.21 mg/g of arsenic respectively. Also the model of Langmuir determined the maximum capacity of adsorption (Qm) of arsenic (As) (III) and (V) by part of raw *E. crassipes*, resulting in 1.1 and 3.21 mg/g of arsenic respectively, increasing almost 4 times more than the maximum capacity of adsorption with this chemical transformation (Lin et al. 2012; Lin et al. 2018).

Through experimentation and modeling with the Langmuir equation, it has been concluded that biochars retain 70.3 mg/g of cadmium (III) per gram of this new adsorbent (Zhang et al. 2015; Feng et al. 2017).

The compound of *E. crassipes*, chitosan, and TiO_2_; the adsorption followed the Langmuir equation; and a maximum adsorption capacity of 256.4 mg/g of Cd (II) were reported for a pH of 7.0 (Chen et al. 2012). The xanthogenase cellulose from *E. crassipes* obtained interesting copper adsorption capacities through the Langmuir isotherm model, retaining 262 mg/g (Tan et al. 2008) and 302 mg/g (Zhou et al. 2011) and 252 mg/g of lead (II) (Deng et al. 2012).

The Freundlich isothermal model is applicable for non-ideal adsorption on heterogeneous and multilayer sorption surfaces (Sastry and Rao 2017; Djehaf et al. 2017; Wanyonyi et al. 2014; Alimohammadi et al. 2017; Mahmoud et al. 2017). Non-linear forms are presented below:

4 $$ \mathrm{qe}=\mathrm{Kf}\ast {\mathrm{Ce}}^{1/n} $$

Moreover, the linear shapes was shown in the following equation:

5 $$ \ln \mathrm{qe}=\mathrm{lnKf}\times \frac{1}{n}\times \mathrm{lnCe} $$

qe: the capacity in the adsorption equilibrium in a given time (mg of metal/g of *E. crassipes*)
Ce: concentrations of heavy metal in equilibrium (mg/L)
Qo: maximum capacity (mg/g of biomass)
KF: indicator of adsorption capacity and *1*/*n* is the intensity of adsorption ((Mg/g) (L mg)/n)

Table 3 shows that the Freundlich isotherm model is the most adequate to describe the adsorption by the biomass of *E. crassipes* on dyes, indicating the heterogeneity of the surface of these biomasses, responsible for the adsorption of multiple layers due to the presence of heterogeneous energy adsorption sites (El-Zawahry et al. 2016; Chisutia and Mmari 2014; Liu et al. 2018; Sumanjit et al. 2015; Singha and Das 2011).

**Table 3:** Freundlich isotherms adjusted in heavy metal treatment with *E. crassipes*

Author	Contaminate	Transformations of biomass	Isoterm of adsortions Freundlich			
			*k*	*n*	*R*^2^	pH
Yi et al. (2016)	Uranio	No	4.013	3.44	0.88	6–7
El-Zawahry et al. (2016)	Colorant		5.666	1.05	0.999	2
Chisutia and Mmari (2014)	Red Congo	No	1.7	0.5	0.923	5
Liu et al. (2018)	Sulfocloropiridazina (SCP)	No	4.82	0.7	0.999	4
Sumanjit et al. (2015)	Colorant	Tensioactivo catiónico y pirolisis de biomasa	1.41	1.64	0.98	7
Singha and Das 2011	Colorant	No	5.6	0.75	0.97	5

This model shows an empirical relationship that does not allow an accurate determination of the adsorption capacity, which is why it is only applied to low and intermediate concentration ranges such as dyes (Muñoz Carpio 2007).

## Kinetics of adsorption of second order

In different studies, it has been found that the adsorption of heavy metals with biomass in phytoremediation processes was adjusted to a second-order model (Mobasherpour et al. 2012; Gao et al. 2010). The pseudo second order model is expressed as:

6 $$ \frac{\mathrm{dq}}{\mathrm{dt}}=\mathrm{K}2{\left(\mathrm{qe}-\mathrm{qt}\right)}^2 $$

When the initial condition is qt = 0, t = 0, the integration of Eq. 6 is:

7 $$ \frac{t}{\mathrm{qt}}=\frac{1}{\mathrm{K}2{\mathrm{qe}}^2}+\frac{t}{\mathrm{qe}}. $$

qe: the capacity in the adsorption equilibrium in a given time (mg of metal/g of *E. crassipes*)
qt: capacity of adsorption in determined time (mg of metal/g of *E. crassipes*)
K2: pseudo second order rate of constant adsorption (g × mg/min)
qe and qt: are the amounts of chromium adsorbed (mg/g) at equilibrium and at time *t* (min), respectively.

The ability to predict the kinetics of heavy metal adsorption is very important for the proper design of a treatment system. Among the available kinetic models, pseudo first order and pseudo second order are the most used to correlate the kinetic absorption data of hazardous substances on raw and treated biomass. In kinetic adsorption studies, the amount of heavy metal adsorbed by the biomass of *E. crassipes* was recorded as a function of time. Through the kinetic profile of adsorption, the type of adsorption mechanism that controls the adsorption process was obtained (Lin et al. 2018).

Most of the kinetic adsorption data conform to a second order model and can be seen in Table 4. Therefore, the main mechanism of control in adsorption using *E. crassipes* as adsorbent is chemisorption; the binding between molecules of heavy metals and functional surface groups of the biomass of *E. crassipes* plays an important role during the adsorption process (Mohanty et al. 2006; Chisutia and Mmari 2014; Liu et al. 2018; Vijetha et al. 2014; Gogoi et al. 2017; Yi et al. 2016; Lin et al. 2012; El-Zawahry et al. 2016; Li et al. 2013; Lin et al. 2018; Ammar et al. 2014; Zhang et al. 2015; Sumanjit et al. 2015; Feng et al. 2017).

**Table 4:** Second order models adjusted in heavy metal treatment with *E. crassipes*

Author	Contaminate	Transformation of biomass	Second order		
			Time of equilibrium	qe(mg/g)	*R*^2^
Yi et al. (2016)	Uranio	No	30	115	0.99
Lin et al. (2012)	Arsenic As (V)	No	50	2.4	0.998
	Arsenic As (III)	No	50	1.1	0.998
El-Zawahry et al. (2016)	Colorant	Cellulose EC	25	0.28	0.9868
Li et al. (2013)	Copper	No	55	13.2	0.999
	Chrome	No	65	13.1	0.885
Chisutia and Mmari (2014)	Red Congo	No	-	5.2	0.9998
Liu et al. (2018)	Sulfocloropiridazina (SCP)	No	-	0.187	0.99
Lin et al. (2018)	Arsenic As (V)	FeOOH	50	9.61	0.993
	Arsenic As (III)	FeOOH	50	5.2	0.998
Ammar et al. (2014)	Cadmium	No	30	3.14	0.99999
	Lead(II)	No	30	3.93	0.9898
Ammar et al. (2014)	Lead	Esferas gelificadas con quitosano	30	7.8	0.9999
Zhang et al. (2015)	Chrome	biochar	500	62	0.992
Sumanjit et al. (2015)	Colorant	Tensioactivo catiónico y pirolisis de biomasa	400	0.00344	0.9998
Feng et al. (2017)	Cadmium	Biochar	450	62	0.9992
Singha and Das (2011)	Colorant	No	-	0.05	0.97
Tan et al. (2008)	Copper	Celulosa xantogenato	20	262	0.93
Zhou et al. (2011)	Copper	Celulosa xantogenato	22	302	0.90
Deng et al. (2012)	Lead	Celulosa xantogenato	25	222	0.88

Table 4 shows the research of Ammar et al. (2014), where they showed that through the second-order kinetic adsorption model, lead (II) was adhered to the gelled spheres of *E. crassipes* and chitosan reaching the equilibrium in 30 min, demonstrating with this model that there was a chemisorption cation exchange. Also, Yi et al. (2016) concluded that the adsorption of U (VI) advanced rapidly with an equilibrium time of 30 min and was satisfied with a kinetic of pseudo second order.

Zhang et al. (2015), in the experimental process of adsorption cadmium by biochar of *E. crassipes*, followed the kinetic model of pseudo second order, reaching equilibrium after 24 h with an initial Cd ranging between 100 and 500 mg/L, concluding that quimiadsorcion is a process by which there was a removal.

Lin et al. (2018) concluded that the removal capacity of *E. crassipes* cellulose with FeOOH forms hydroxides that are responsible for the cation exchange with arsenic (V). This process was modeled through the second order model, determining a cationic exchange of chemisorption, and with this model, the equilibrium capacity of 20 min was determined with initial concentration of 100 mg/L of As (V).

## Design of treatment systems

In order to design different biological filters, different adsorption capacities of several biomass materials of chemically modified *E. crassipes* were studied, and kinetic studies was carried out, determining the saturation times of each biomass. With these parameters, we will proceed to design an economical and efficient biofilter when it comes to retaining heavy metals.

Biofilters are industrial wastewater treatment systems contaminated mainly by dyes and heavy metals; they are composed of a bioreactor, where it stores biological material (plant, fungus, or bacteria) (Lesmana et al. 2009; Rengel 2018). The main variables in the design of filters proposed by Tchobanoglous (2000) are:

- Characteristics of the filter medium (biomass of *E. crassipes*).
- Porosity of the bed (biomass of *E. crassipes*).
- Depth of the bed (biomass of *E. crassipes*).
- Filtration rate (adsorption capacity).
- Saturation of the (biomass of *E. crassipes*).
- Characteristics of the tributary.

The main components of a biofilter are the regulating tank, regulating valves, input devices, biomass, base, and sampling, based on Sarkar et al. (2017). Biofilters with red algae *Chondracanthus* *chamissoi* (Macassi 2014) and *Lantama camara* (Rengel 2018) with cane bagasse (Macassi 2014) have been designed and constructed.

**Fig. 9:**
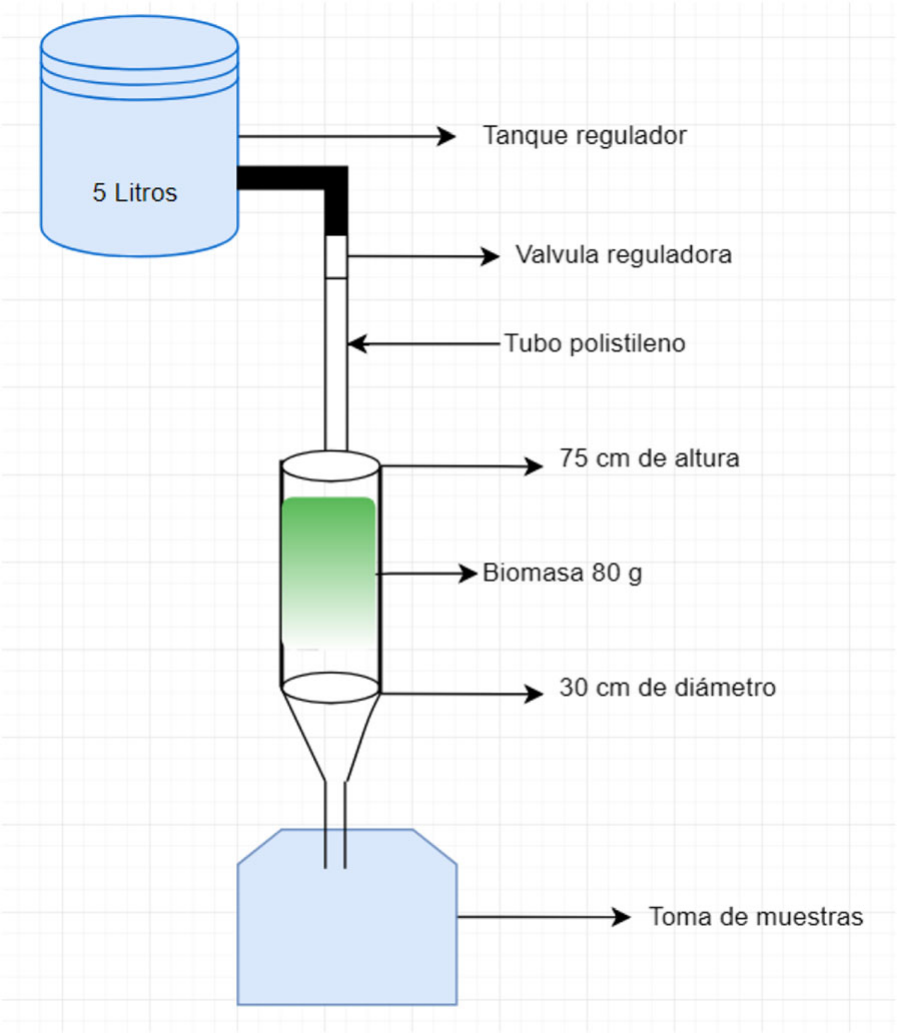
Proposed design for the filter (based on Sarkar et al. 2017)

Filters with ground *E. crassipes* have been designed (see Fig. 9) using about 20 g of dry biomass, and from this plant, with a vertical descending flow treatment, 5 l of contaminated water from a tannery were treated, removing about 70% chromium (VI), with initial concentrations of 200 mg/L (Sarkar et al. 2017).

With the models of adsorption kinetics, (seen in chapter 4) where the equilibrium time of the biomass of *E. crassipes* was obtained and with the model of isotherms of adsorption of Langmuir where the maximum capacity was obtained (seen in chapter 3) of the biomass to retain heavy metals and dyes, it proposed in this article some designs based on these two models to treat water on an industrial scale.

The designs will be adapted to the one proposed by Sarkar et al. (2017), where the treatment column would be made of a reactor of 30 cm of internal diameter and 150 cm of height, where the treatments should be carried out with a vertical downward flow.

Table 5 shows the different types of *E. crassipes* biomass transformed with the heavy metal adsorption capacities and the costs associated with this treatment system to treat a flow of 5 l of contaminant. In addition, this table summarizes six different biomass of *E. crassipes* to remedy some types of contaminated water from different industries. Next, each process is described, adapting to the needs of the contaminated effluents.

1. The case study is that of a small tannery in the south of the city of Bogotá; this tannery has a flow rate of 5 l/day of water contaminated with chromium (VI), with a concentration of 150 mg/L. The treatment ideal is cellulose xanthogenate, according to Tan et al. (2008); this cellulose has a capacity to remove 232 mg/g of chromium (VI), retaining 90% of the initial concentration, where it would take 20 g of *E. crassipes* and 15 g of NaOH, 2 ml of Cs2, and 5 g of MgSO4. It was estimated that the total cost of this treatment system is 55 dollars and the replacements of the biomass have a cost of 15 dollars each month.
2. Arsenic was generated in the industrial process of glass, pigments, textiles, paper, metal adhesives, wood protectors, ammunition, among others, for this type of polluted water; this industry generates a total of 6 L/day of water contaminated with 100 mg/L of arsenic (V). To treat this water, the biomass transformed by Lin et al. (2018) is propos, since it can retain 9.21 mg of As (V), per gram of biomass, it would need a treatment system with 50 g of biomass of *E. crassipes* and 400 g of iron chloride to prepare 50 g of EC FeOOH; this treatment system would cost $100 and $10 each month in the replacements of the biomass. The modification of the iron in the biomass of *E. crassipes* reduces the amount of dry biomass of this plant, since in the treatment of raw biomass it would take almost 250 g to retain the same amount of arsenic, increasing costs and time of treatment.
3. The mining industry throws large concentrations of lead and mercury; a system with gelled spheres of chitosan and *E. crassipes* is propos for its treatment. According to Ammar et al. (2014), these spheres retain 321 mg/g heavy metal per gram of gelled biomass, requiring a treatment system with 25 g of biomass to treat 10 L with concentrations of 100 mg/L of both mercury and lead, with a cost of $80 for the construction of this treatment system and $15 each month in the replacement of biomass.
4. In the treatment of contaminated effluents from a textile industry, the biomass worked by El-Zawahry et al. (2016) was proposed, where they created a network composed of cellulose of *E. crassipes*, chitosan, and titanium oxide (TiO). This treatment would have removals above 98% Congo red as a function of pH, where a treatment system would be needed with 25 g of biomass of *E. crassipes* and 25 g of chitosan, together with 1 L of titanium oxide. These materials would cost $100 and $15 each month in replacing the biomass, a bit more expensive due to the cost of titanium oxide (TiO).
5. In the paint manufacturing industry, large amounts of cadmium (Cd) are spilled; for this water, the biochar of *E. crassipes* proposed by Zhang et al. (2015) is proposed; with the model of Lagmuir, the amount of 35 was determined. Grams of biochar to treat 5 l of contaminated water with initial cadmium concentration of 120 mg/L has a cost of $120 and $75 each month in the replacements of the biomass, compared with the other treatment systems, one of the most expensive due to energy expenditure.
6. For the treatment of oil spills, aerogels with *E. crassipes* and polyvinyl alcohol created by Yin et al. (2017) was used; it would be necessary, unlike the other oven treatments and a lyophilizer, increasing the costs of their treatment; it takes 25 g of biomass of *E. crassipes* and 10 L of polyvinyl alcohol. These materials would cost $4500 to treat about 10 l.

**Table 5:** Treatment design on a larger-scale biomass transformed from *E. crassipes*

Biomass of *E. crassipes* +	Industry	Treatment L/day	Concentration initial, contaminate mg/L	Price of treatment (dollars)/implementation	Author
Cellulose xantogenato	Tenneries	5	150 Cr(V)	55	Tan et al. 2008
Cellulose FeOOH	Papers	10	100 As(V)	100	Lin et al. 2018
Spheres	Minery	10	100 Pb (II)	80	Ammar et al. 2014
Biochar	Pains	10	120 Cd	1200	Zhang et al. 2015
Nanoparticles	Textile	5	150 Congo red	100	El-Zawahry et al. 2016
Gels-cellulose	Oil spills	10	100 Aceite en agua	1000	Yin et al. (2017)

## Discussion and conclusions

Colombia is the country more contaminated with mercury of latinoamerica, due the conflicted armaded of very years. This biotechnology is one solutions economic and efficient for treatment of water contaminated with different heavy metals.

The design of biofilter would have one characterization of water that can develop the system treatment viability. It recommended the use xanthogenato of the cellulose of *E. crassipes* for treatment of water contaminated with chromium. For the treatment of water contaminated with arsenic, it is a must to develop the biofilter with biomass of *E. crassipes* transformed with iron. In the treatment of contaminated effluents from a textile, industry would develop the biofilter with *E. crassipes*, chitosan, and titanium oxide (TiO).

The mechanism of adsorption of the biomass of the *E. crassipes* about heavy metals and colorant is very efficient, and this must make transformation chemistry for the optimizing of treatment.

The maximum capacity found through the isothermal models was fundamental to propose designs on a larger scale of treatment of waters contaminated with heavy metals, since it provides the ideal capacity of metal per gram of biomass of *E. crassipes*.

The equilibrium capacities were determined through second order models, finding that the chemisorption is the mechanism that most adjusts between the biomass of *E. crassipes* and different heavy metals in the water.

Large-scale designs of water treatment, contaminated with heavy metals and dyes, must be developed and executed, as there is much research, but none of them is about treating water on a larger scale.

## References

[CR1] Abdel-Fattah AF, Abdel-Naby MA (2012). Pretreatment and enzymic saccharification of water hyacinth cellulose. Carbohydrate Polymers.

[CR2] Adornado AP, Soriano AN, Orfiana ON, Pangon MBJ, Nieva AD (2017). Simulated biosorption of Cd (II) and Cu (II) in single and binary metal systems by water hyacinth (Eichhornia crassipes) using Aspen adsorption. ASEAN Journal of Chemical Engineering.

[CR3] Ajitha, P., Vijayalakshmi, K., Saranya, M., Gomathi, T., Rani, K., Sudha, P. N., & Sukumaran, A. (2017). Removal of toxic heavy metal lead (II) using chitosan oligosaccharide-graft-maleic anhydride/polyvinyl alcohol/silk fibroin composite. *International journal of biological macromolecules, 104*, 1469–1482. 10.1016/j.ijbiomac.2017.05.111 28539265

[CR4] Alimohammadi V, Sedighi M, Jabbari E (2017). Estudio experimental en la eliminación eficaz de hierro total de las aguas residuales utilizando nanotubos de carbono de múltiples paredes magnéticas modificado. Ingeniería ecológica.

[CR5] Ammar NS, Elhaes H, Ibrahim HS, Ibrahim MA (2014). A novel structure for removal of pollutants from wastewater. Spectrochimica Acta Part A: Molecular and Biomolecular Spectroscopy.

[CR6] Anirudhan TS, Shainy F (2015). Adsorption behaviour of 2-mercaptobenzamide modified itaconic acid-grafted-magnetite nanocellulose composite for cadmium (II) from aqueous solutions. Journal of Industrial and Engineering Chemistry.

[CR7] Anirudhan TS, Deepa JR, Christa J (2016). Nanocellulose/nanobentonite composite anchored with multi-carboxyl functional groups as an adsorbent for the effective removal of cobalt (II) from nuclear industry wastewater samples. Journal of Colloid and Interface Science.

[CR8] Ariff, M., Farhana, N., Hanafiah, M., Kamal, M. A., Ngah, W., & Saime, W. (2017). La adsorción de Cu (II) sobre recubierto de quitosano perlas de bentonita reticulados: Los estudios cinéticos y de isotermas. En clave Materiales de Ingeniería (Vol. 753, pp. 243-248). Trans Tech Publications.

[CR9] Atehortua, E., & Gartner, C. (2003). Preliminary studies of Eichhornia crassipes dry biomass for lead and chromium removal from waters. *Revista Colombiana de Materiales, 5*.

[CR10] Balasubramaniana, K., Arunachalama, A., Dasb, K., & Arunachalama. (2012). *Decomposition and nutrient release of Eichhornia crassipes (Mart.) Solms. Under different trophic conditions in wetlands of eastern Himalayan foothills., Ecological Engineering*, 2012.

[CR11] Baldeón, L. Q., Chavez, J. B. A., Suarez, C. F. M., & Huaranga, M. C. (2017). Eficiencia de la especie macrófita Eichhornia crassipes (Jacinto de agua) para la remoción de parámetros fisicoquímicos, metal pesado (Pb) y la evaluación de su crecimiento en función al tiempo y adopción al medio en una laguna experimental. *Revista de Investigación Ciencia, Tecnología y Desarrollo, 1*(1).

[CR12] Baldikova E, Pospiskova K, Ladakis D, Kookos IK, Koutinas AA, Safarikova M, Safarik I (2017). Magnetically modified bacterial cellulose: a promising carrier for immobilization of affinity ligands, enzymes, and cells. Materials Science and Engineering: C.

[CR13] Borker AR, Mane AV, Saratale GD, Pathade GR (2013). Phytoremediation potential of Eichhornia crassipes for the treatment of cadmium in relation with biochemical and water parameters. Emirates Journal of Food and Agriculture.

[CR14] Bronzato, G. R. F. (2016). Investigação da biomassa de Eichhornia crassipes (aguapé) para a obtenção de etanol de segunda geração como um processo mitigatório da poluição aquática.

[CR15] Chen, A., Zeng, G., Chen, G., Hu, X., Yan, M., Guan, S., et al. (2012). Novel thiourea-modified magnetic ion-imprinted chitosan/TiO2 composite for simultaneous removal of cadmium and 2, 4-dichlorophenol. *Chemical Engineering Journal, 191*, 85–94.

[CR16] Chitpong, N., & Husson, S. M. (2017). Polyacid functionalized cellulose nanofiber membranes for removal of heavy metals from impaired waters. *Journal of Membrane Science, 523*, 418–429.

[CR17] Chisutia W, Mmari O (2014). Adsorption of Congo red dye from aqueous solutions using roots of Eichhornia crassipes: kinetic and equilibrium studies. Energyprocedia.

[CR18] Chuang YS, Lay CH, Sen B, Chen CC, Gopalakrishnan K, Wu JH (2012). Biohydrogen and biomethane from water hyacinth (*Eichhornia crassipes*). International Journal of Hydrogen Energy.

[CR19] Crini G (2005). Recent developments in polysaccharide-based materials used as adsorbents in wastewater treatment. Progress in Polymer Science.

[CR20] Deng L, Geng M, Zhu D, Zhou W, Langdon A, Wu H, Yu Y, Zhu Z, Wang Y (2012). Effect of chemical and biological degumming on the adsorption of heavy metal by cellulose xanthogenates prepared from Eichhornia crassipes. Bioresource Technology.

[CR21] Dhankhar, R., & Hooda, A. (2011). Fungal biosorption–an alternative to meet the challenges of heavy metal pollution in aqueous solutions. *Environmental technology, 32*(5), 467–491. 10.1080/09593330.2011.572922 21877528

[CR22] Djehaf K, Bouyakoub AZ, Ouhib R, Benmansour H, Bentouaf A, Mahdad A (2017). aguas residuales textiles en Tlemcen (Argelia occidental): Impacto, Tratamiento por proceso combinado. Química International.

[CR23] El-Zawahry MM, Abdelghaffar F, Abdelghaffar RA, Hassabo AG (2016). Equilibrium and kinetic models on the adsorption of reactive black 5 from aqueous solution using Eichhornia crassipes/chitosan composite. Carbohydrate Polymers.

[CR24] Feng W, Xiao K, Zhou W, Zhu D, Zhou Y, Yuan Y, Xiao N, Wan X, Hua Y, Zhao J (2017). Analysis of utilization technologies for Eichhornia crassipes biomass harvested after restoration of wastewater. Bioresource Technology.

[CR25] Ganguly A, Chatterjee PK, Dey A (2012). Studies on ethanol production from water hyacinth—a review. Renewable and Sustainable Energy Reviews.

[CR26] Gao J, Zhang Q, Su K, Chen R, Peng Y (2010). Biosorción de Acid Yellow 17 a partir de solución acuosa por el lodo granular no viviente aeróbico. Diario de materiales peligrosos.

[CR27] Gogoi, P., Adhikari, P., & Maji, T. K. (2017). La biorremediación de arsenic del agua con polvo de raíz de reticulado de ácido cítrico jacinto de agua (*E. crassipes*). monitoreo y evaluación ambientales.*, 189*(8), 383.

[CR28] Gupta A, Balomajumder C (2015). Removal of Cr (VI) and phenol using water hyacinth from single and binary solution in the artificial photosynthesis chamber. Journal of Water Process Engineering.

[CR29] Hadad HR, Maine MA, Mufarrege MM, Del Sastre MV, Di Luca GA (2011). Bioaccumulation kinetics and toxic effects of Cr, Ni and Zn on Eichhornia crassipes. Journal of Hazardous Materials.

[CR30] Hokkanen S, Repo E, Sillanpää M (2013). Removal of heavy metals from aqueous solutions by succinic anhydride modified mercerized nanocellulose. Chemical Engineering Journal.

[CR31] Hokkanen S, Repo E, Westholm LJ, Lou S, Sainio T, Sillanpää M (2014). Adsorption of Ni2+, Cd2+, PO43− and NO3− from aqueous solutions by nanostructured microfibrillated cellulose modified with carbonated hydroxyapatite. Chemical Engineering Journal.

[CR32] Hokkanen S, Repo E, Lou S, Sillanpää M (2015). Removal of arsenic (V) by magnetic nanoparticle activated microfibrillated cellulose. Chemical Engineering Journal.

[CR33] Huang X, Zhan X, Wen C, Xu F, Luo L (2018). Amino-functionalized magnetic bacterial cellulose/activated carbon composite for Pb2+ and methyl orange sorption from aqueous solution. Journal of Materials Science & Technology.

[CR34] Ibrahim HS, Ammar NS, Soylak M, Ibrahim M (2012). Removal of Cd (II) and Pb (II) from aqueous solution using dried water hyacinth as a biosorbent. Spectrochimica Acta Part A: Molecular and Biomolecular Spectroscopy.

[CR35] Jin X, Xiang Z, Liu Q, Chen Y, Lu F (2017). Polyethyleneimine-bacterial cellulose bioadsorbent for effective removal of copper and lead ions from aqueous solution. Bioresource Technology.

[CR36] Kobielska PA, Howarth AJ, Farha OK, Nayak S (2018). Metal–organic frameworks for heavy metal removal from water. Coordination Chemistry Reviews.

[CR37] Kumar R, Sharma RK, Singh AP (2017). Cellulose based grafted biosorbents-journey from lignocellulose biomass to toxic metal ions sorption applications-a review. Journal of Molecular Liquids.

[CR38] Lay B, Sen CC, Chen JH, Wu SC, Lee CYL (2013). Co-fermentation of water hycianth and beverage wastewater in powder and pellet form for hydrogen production. Bioresource Technology.

[CR39] Lesmana, S. O., Febriana, N., Soetaredjo, F. E., Sunarso, J., & Ismadji, S. (2009). Studies on potential applications of biomass for the separation of heavy metals from water and wastewater. *Biochemical Engineering Journal, 44*(1), 19–41.

[CR40] Li X, Liu S, Na Z, Lu D, Liu Z (2013). La adsorción, la concentración, y la recuperación de iones de metales pesados acuosas con el polvo de raíz de Eichhornia crassipes. Ingeniería ecológica.

[CR41] Lin K, Pan J, Chen Y, Cheng R, Xu X (2009). Estudio de la adsorción de fenol a partir de solución acuosa en nanopolvos de hidroxiapatita. Diario de materiales peligrosos.

[CR42] Lin S, Wang G, Na Z, Lu D, Liu Z (2012). Long-root Eichhornia crassipes as a biodegradable adsorbent for aqueous as (III) and as (V). Chemical Engineering Journal.

[CR43] Lin S, Yang H, Na Z, Lin K (2018). A novel biodegradable arsenic adsorbent by immobilization of iron oxyhydroxide (FeOOH) on the root powder of long-root Eichhornia crassipes. Chemosphere.

[CR44] Liu L, Hu S, Shen G, Farooq U, Zhang W, Lin S, Lin K (2018). Adsorption dynamics and mechanism of aqueous sulfachloropyridazine and analogues using the root powder of recyclable long-root Eichhornia crassipes. Chemosphere.

[CR45] Lu K, Yang X, Gielen G, Bolan N, Ok YS, Niazi NK (2017). Effect of bamboo and rice straw biochars on the mobility and redistribution of heavy metals (Cd, Cu, Pb and Zn) in contaminated soil. Journal of Environmental Management.

[CR46] Macassi AL (2014). Diseño de un biofiltro a base del alga roja cochayuyo (Chondracanthus Chamissoi) para la remoción de cromo de efluentes de la industria del curtido.

[CR47] Magdum SM, More, Nadaf AA (2012). Biochemical conversion of acid pretreatment water hyacinth (eichonnia crassipes) to alcohol using pichia stipitis NCIM 3497. International Journal of Advanced Biotechnology and Research.

[CR48] Mahmoud MS, Mostafa MK, Mohamed SA, Sobhy NA, Nasr M (2017). La biorremediación de colorante azo rojo de las soluciones acuosas por la cepa de Aspergillus niger aislada de las aguas residuales textiles. Revista de Ingeniería Química Ambiental.

[CR49] Mahunon, S. E. R., Aina, M. P., Akowanou, A. V. O., Kouassi, E. K., Yao, B. K., Adouby, K., & Drogui, P. (2018). Optimization process of organic matter removal from wastewater by using Eichhornia crassipes. *Environmental Science and Pollution Research*, 1–8. 10.1007/s11356-018-2771-y 30117026

[CR50] Martínez C, Torres LM, de la Cruz RFG (2013). Evaluación de la cinética de adsorción de Zn2 + y Cd2 + a partir de soluciones binarias Unitarias y por Eichhornia crassipes Raíces de Typha latifolia y. Avances en Ciencias e Ingeniería.

[CR51] Mishima D, Kuniki M, Sei B, Soda, Ike M, Fujita M (2008). Ethanol production from candidate energy crops: Water hyacinth (Eichhornia crassipes) and water lettuce (Pistia stratiotes L.). Bioreosur Tecnhol..

[CR52] Mobasherpour I, Salahi E, Pazouki M (2012). Comparativo de la eliminación de Pb2 +, Cd2 + y Ni2 + por hidroxiapatita cristalito nano partir de soluciones acuosas: estudio isoterma de adsorción. Diario de Química Arabian.

[CR53] Mohammed AB, Omran AR, Baiee MA, Salman JM (2018). Biosorption of Safranin-O from aqueous solution by Nile Rose Plant (Eichhornia crassipes). Baghdad Science Journal.

[CR54] Mohanty K, Jha M, Meikap B, Biswas M (2006). Biosorption of Cr(VI) from aqueous solutions by Eichhornia crassipes. Chemical Engineering Journal.

[CR55] Muñoz Carpio JC (2007). Biosorción de lead(II) por cáscara de naranja “citrus cinensis” pretratada.

[CR56] Nagarajan, D., Lee, D. J., Kondo, A., & Chang, J. S. (2017). Recent insights into biohydrogen production by microalgae–from biophotolysis to dark fermentation. *Bioresource technology, 227*, 373–387. 10.1016/j.biortech.2016.12.104. 10.1016/j.biortech.2016.12.104 28089136

[CR57] Niu Y, Li K, Ying D, Wang Y, Jia J (2017). Novel recyclable adsorbent for the removal of copper (II) and lead (II) from aqueous solution. Bioresource Technology.

[CR58] Nguyen, T. C., Loganathan, P., Nguyen, T. V., Kandasamy, J., Naidu, R., & Vigneswaran, S. (2018). Adsorptive removal of five heavy metals from water using blast furnace slag and fly ash. *Environmental Science and Pollution Research, 25*(21), 20430–20438. 10.1007/s11356-017-9610-4 28707235

[CR59] Park D, Yun YS, Jo JH, Park JM (2005). Mechanism of hexavalent chromium removal by dead fungal biomass of Aspergillus niger. Water Research.

[CR60] Park D, Yun YS, Park JM (2006). Mechanisms of the removal of hexavalent chromium by biomaterials or biomaterial-based activated carbons. Journal of Hazardous Materials.

[CR61] Park JH, Anburajan P, Kumar G, Park HD, Kim SH (2017). Biohydrogen production integrated with an external dynamic membrane: a novel approach. International Journal of Hydrogen Energy.

[CR62] Pillai SS, Deepa B, Abraham E, Girija N, Geetha P, Jacob L, Koshy M (2013). Biosorption of Cd (II) from aqueous solution using xanthated nano banana cellulose: equilibrium and kinetic studies. Ecotoxicology and Environmental Safety.

[CR63] Putro JN, Kurniawan A, Ismadji S, Ju YH (2017). Nanocellulose based biosorbents for wastewater treatment: study of isotherm, kinetic, thermodynamic and reusability. Environmental Nanotechnology, Monitoring & Management.

[CR64] Putro JN, Santoso SP, Ismadji S, Ju YH (2017). Investigation of heavy metal adsorption in binary system by nanocrystalline cellulose–bentonite nanocomposite: improvement on extended Langmuir isotherm model. Microporous and Mesoporous Materials.

[CR65] Rahchamani J, Mousavi HZ, Behzad M (2011). Adsorption of methyl violet from aqueous solution by polyacrylamide as an adsorbent: isotherm and kinetic studies. Desalination.

[CR66] Rahman SNA, Masdar MS, Rosli MI, Majlan EH, Husaini T, Kamarudin SK, Daud WRW (2016). Overview biohydrogen technologies and application in fuel cell technology. Renewable and Sustainable Energy Reviews.

[CR67] Rani N, Singh B, Shimrah T (2017). Chromium (VI) removal from aqueous solutions using Eichhornia as an adsorbent. Journal of Water Reuse and Desalination.

[CR68] Rengel Calvopiña, M. M. (2018). Evaluación de la capacidad de adsorción de la supirrosa (Lantana camara) en la remoción de cromo de aguas residuales de la industria de curtiembre(Bachelor's thesis, Quito, 2018.).

[CR69] Ri PC, Ren NQ, Ding J, Kim JS, Guo WQ (2017). CFD optimization of horizontal continuous stirred-tank (HCSTR) reactor for bio-hydrogen production. International Journal of Hydrogen Energy.

[CR70] Ruan C, Strømme M, Lindh J (2016). A green and simple method for preparation of an efficient palladium adsorbent based on cysteine functionalized 2, 3-dialdehyde cellulose. Cellulose.

[CR71] Saman N, Johari K, Song ST, Kong H, Cheu SC, Mat H (2017). High removal efficacy of hg (II) and MeHg (II) ions from aqueous solution by organoalkoxysilane-grafted lignocellulosic waste biomass. Chemosphere.

[CR72] Saraswat S, Rai JPN (2010). Heavy metal adsorption from aqueous solution using Eichhornia crassipes dead biomass. International Journal of Mineral Processing.

[CR73] Sarkar M, Rahman AKML, Bhoumik NC (2017). Remediation of chromium and copper on water hyacinth (E. crassipes) shoot powder. Water Resources and Industry.

[CR74] Sastry, S. V. A. R., & Rao, B. (2017). Determinación de la cinética de adsorción para la eliminación de copper a partir de agua residual, usando pasó extracto de té (STE). *Diario sobre la futura Ingeniería y Tecnología, 12*(4).

[CR75] Singha B, Das SK (2011). Biosorption of Cr (VI) ions from aqueous solutions: kinetics, equilibrium, thermodynamics and desorption studies. Colloids and Surfaces B: Biointerfaces.

[CR76] Stoica-Guzun A, Stroescu M, Jinga SI, Mihalache N, Botez A, Matei C, Berger D, Damian CM, Ionita V (2016). Box-Behnken experimental design for chromium (VI) ions removal by bacterial cellulose-magnetite composites. International Journal of Biological Macromolecules.

[CR77] Sumanjit K, Rani S, Mahajan RK, Asif M, Gupta VK (2015). Synthesis and adsorption properties of mesoporous material for the removal of dye safranin: kinetics, equilibrium, and thermodynamics. Journal of Industrial and Engineering Chemistry.

[CR78] Swain, G., Adhikari, S., & Mohanty, P. (2014). Phytoremediation of copper and cadmium from water using water hyacinth, *Eichhornia crassipes*. *International Journal of Agricultural Science and Technology*.

[CR79] Tan L, Zhu D, Zhou W, Mi W, Ma L, He W (2008). Preferring cellulose of *Eichhornia crassipes* to prepare xanthogenate to other plant materials and its adsorption properties on copper. Bioresource Technology.

[CR80] Tchobanoglous, C. R. I. T. E. S. Y. (2000). *Tratamiento de Aguas Residuales en Pequeñas Poblaciones* (776p). S.A. Santafé de Bogotá: McGraw-Hill Interamericana.

[CR81] Tortora F, Innocenzi V, De Michelis I, Vegliò F, di Celso GM, Prisciandaro M (2018). Recovery of anionic surfactant through acidification/ultrafiltration in a micellar-enhanced ultrafiltration process for cobalt removal. Environmental Engineering Science.

[CR82] Vijetha P, Kumaraswamy K, Kumar YP, Satyasree N, Prasad KS (2014). Biosorption of Cu, Zn and Pb by Eichhornia crassipes: thermodynamic and isotherm studies. International Journal of Scientific & Technology Research.

[CR83] Wang CY, Sample DJ, Day SD, Grizzard TJ (2015). Floating treatment wetland nutrient removal through vegetation harvest and observations from a field study. Ecological Engineering.

[CR84] Wang J, Lu X, Ng PF, Lee KI, Fei B, Xin JH, Wu JY (2015). Polyethylenimine coated bacterial cellulose nanofiber membrane and application as adsorbent and catalyst. Journal of Colloid and Interface Science.

[CR85] Wang C, Wang H, Gu G (2018). Ultrasound-assisted xanthation of cellulose from lignocellulosic biomass optimized by response surface methodology for Pb (II) sorption. Carbohydrate Polymers.

[CR86] Wanyonyi WC, Onyari JM, Shiundu PM (2014). La adsorción de colorante Rojo Congo a partir de soluciones acuosas utilizando raíces de Eichhornia crassipes: estudios cinéticos y de equilibrio. Energía Procedia.

[CR87] Wei Y, Fang Z, Zheng L, Tsang EP (2017). nanopartículas de hierro biosintetizada en extractos acuosos de Eichhornia crassipes y su mecanismo en la eliminación de chrome hexavalente. Superficie Applied Science.

[CR88] Wu H, Zhang J, Ngo HH, Guo W, Hu Z, Liang S, Fan J, Liu H (2015). A review on the sustainability of constructed wetlands for wastewater treatment: design and operation. Bioresource Technology.

[CR89] Yap MW, Mubarak NM, Sahu JN, Abdullah CE (2017). síntesis inducida por microondas de biocarbón magnético a partir de biomasa agrícola para la eliminación de leady cadmiumde las aguas residuales. Journal of Industrial and Engineering Chemistry.

[CR90] Yi ZJ, Yao J, Chen HL, Wang F, Yuan ZM, Liu X (2016). Uranium biosorption from aqueous solution onto Eichhornia crassipes. Journal of Environmental Radioactivity.

[CR91] Yin T, Zhang X, Liu X, Wang C (2017). Resource recovery of Eichhornia crassipes as oil superabsorbent. Marine Pollution Bulletin.

[CR92] Yu X, Tong S, Ge M, Wu L, Zuo J, Cao C, Song W (2013). Adsorption of heavy metal ions from aqueous solution by carboxylated cellulose nanocrystals. Journal of Environmental Sciences.

[CR93] Zhang F, Wang X, Yin D, Peng B, Tan C, Liu Y, Tan X, Wu S (2015). Efficiency and mechanisms of Cd removal from aqueous solution by biochar derived from water hyacinth (Eichornia crassipes). Journal of Environmental Management.

[CR94] Zhang N, Zang GL, Shi C, Yu HQ, Sheng GP (2016). A novel adsorbent TEMPO-mediated oxidized cellulose nanofibrils modified with PEI: preparation, characterization, and application for Cu (II) removal. Journal of Hazardous Materials.

[CR95] Zhou W, Zhu D, Langdon A, Li L, Liao S, Tan L (2009). The structure characterization of cellulose xanthogenate derived from the straw of Eichhornia crassipes. Bioresource Technology.

[CR96] Zhou W, Ge X, Zhu D, Langdon A, Deng L, Hua Y, Zhao J (2011). Metal adsorption by quasi cellulose xanthogenates derived from aquatic and terrestrial plant materials. Bioresource Technology.

[CR97] Zhou C, Wu Q, Lei T, Negulescu II (2014). Adsorption kinetic and equilibrium studies for methylene blue dye by partially hydrolyzed polyacrylamide/cellulose nanocrystal nanocomposite hydrogels. Chemical Engineering Journal.

